# Fermented fruits: scrumping, sharing, and the origin of feasting

**DOI:** 10.1093/biosci/biaf102

**Published:** 2025-07-31

**Authors:** Nathaniel J Dominy, Luke D Fannin, Erin R Vogel, Martha M Robbins, Catherine Hobaiter

**Affiliations:** Department of Anthropology, Department of Biological Sciences, Dartmouth College, Hanover, New Hampshire, United States; Department of Anthropology, Graduate Program in Ecology, Evolution, Environment, Society, Dartmouth College, Hanover, New Hampshire, United States; Department of Anthropology, Center for Human Evolution Studies, Rutgers, the State University of New Jersey, New Brunswick, New Jersey, United States; Department of Primate Behavior and Evolution, Max Planck Institute for Evolutionary Anthropology, Leipzig, Germany; School of Psychology and Neuroscience, University of St Andrews, St Andrews, Scotland, United Kingdom

## Abstract

Mounting evidence points to the importance of fermented fruits in the diets of tropical frugivores, especially African apes. But how has this fundamental aspect of ape ecology escaped scientific attention over the past six decades? Here we draw inspiration from the Middle Ages to fill an essential void in scientific discourse.

The quality, availability, and accessibility of fruit are integral to almost every aspect of ape biology and behavior, and it is practically axiomatic to describe apes as frugivores. It is a classification that rings true even when fruit is scarce, because all apes show a categorical preference for ripening fruit when available, a behavior that holds renewed significance in light of a recent review by Bowland et al. (2025a). Their work puts a spotlight on the underrecognized role of dietary ethanol as a selective force on tropical frugivores, and it shows that most ripening fruit is fermented to some degree. Welcome and timely, their paper raises tantalizing questions on the ethanol content and ecology of fallen fruits, a topic with special relevance for human evolution (Dudley and Maro 2021).

Carrigan et al. (2015) drew attention to fallen fruits in the diets of African apes. Their study reported the protein sequences and corresponding kinetic activities of alcohol dehydrogenase class IV (ADH4), the first enzyme to encounter and metabolize dietary ethanol. They sequenced the genes of 18 primate species and resurrected nine ancestral proteins to trace the evolution and functional ecology of ADH4. This innovative approach produced two important findings. First, the ADH4 enzymes of most primates are essentially inactive against ethanol. Second, a single amino acid change (A294V) in the gene of the last common ancestor of African apes accounts for a dramatic fortyfold increase in ethanol-catalyzing activity. The significance of this mutation is rather profound, representing, potentially, a signal moment in the history of life on Earth. If the early domestication of cereals revolved around making beer instead of bread, then the A294V mutation was the preadaptation that fueled the Neolithic Revolution and everything that followed (Dominy 2015).

## Happy hour

To explain the retention of this mutation, Carrigan et al. (2015) argued that increasing terrestrialism during the Middle Miocene exposed the ancestor of crown African apes to “overripe [fermented] fruit that [had] fallen to the ground” (p. 461). It is a compelling idea that suffers from two problems: the ethanol exposure of African apes is all but unknown, and primatologists seldom differentiate fallen fruits from arborescent ones in their field notes, meaning we have little sense of how often apes consume fruits from the ground. In fact, we don't even have a word for this behavior.

“The history of ideas is paved by constraints of language”

—Leo W. Buss in *The Evolution of Individuality* (1988).

Nobody wants more jargon, but sometimes we need a new word, or neologism, to capture a fundamental concept. Albert Bernhard Frank coined the word *symbiosis* in 1877 to describe the astonishing mutualisms of lichens (Martin and Schwab 2012), and it is difficult to imagine pop culture today without the word *meme*, a gift of Richard Dawkins in 1976 (Dawkins 1976). In primatology, we didn't know how much we needed the word *cathemeral* (to describe the arrhythmic activity patterns of some lemurs) until lan Tattersall invented it in 1988 (Tattersall 2006). New words can propel scientific discourse and discovery, and we wondered whether progress on the topic of fruit–animal interactions has been stymied for want of language. So we puzzled over how to reduce *consumption-of-fallen-fruit* into a single practical word.

## Scrumping

Scrumping is the act of gathering—or sometimes stealing—windfallen apples and other fruit. It is an English derivation of the Middle Low German word *schrimpen* (“shriveled, shrunken”), a medieval noun for describing overripe or fermented fruit. It is an obscure origin perhaps, but its legacy echoes in many British pubs today, where patrons can order scrumpy, a cloudy apple cider with an alcohol by volume (ABV) content that ranges between 6 and 9%. The adjective *scrumptious* (something delicious, alluring) alludes to fruit and temptation, a frequent motif in Gothic art and architecture. Indeed, the Gothic tradition recognized scrumping as an essential behavior of nonhuman primates (box 1).

Box 1. **Scrumping monkeys in the Garden of Eden**

**Figure:**
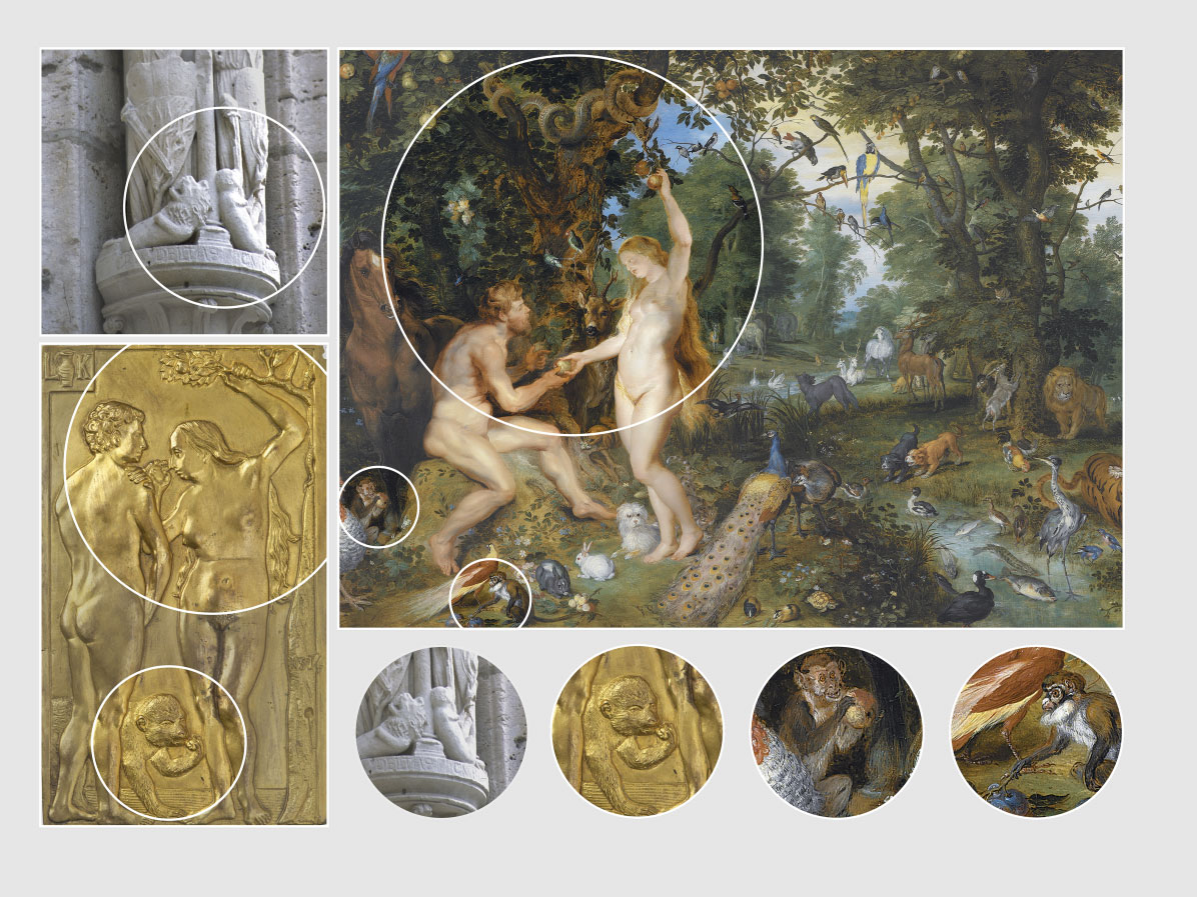
Gothic monkeys as the embodiment of curiosity and temptation. Top left: a column statue, Cathédrale de Chartres. At left, Fortitude stabs a lion (inscription *crudelitas*, “cruelty”); at right, Justice conquers a monkey (inscription *curiositas*, “curiosity”). Photograph: Jane Vadnal, reproduced with permission. Bottom left: a bronze plaquette with gilding titled *Adam and Eve*, dated 1514 by Ludwig Krug. Photograph: Hood Museum of Art, Dartmouth College, reproduced with permission. Right: panel painting titled *Earthly Paradise with the Fall of Man*, dated 1615 by Peter Paul Rubens (the figures) and Jan Brueghel the Elder (the flora and fauna, including two monkeys resembling *Cercocebus torquatus* on the left and *Cercopithecus petaurista* on the right). Photograph reproduced under CC0 1.0 license.



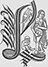
ustful, fruit-eating monkeys were an influential motif during the Middle Ages. Their origin can be traced to twelfth century bestiaries and the *Mater Verborum*, an encyclopaedic dictionary. Both works featured monkeys handling fruit, a Biblical symbol of temptation. By 1499, this iconography expanded to include Eve, who appeared, alongside a fruit-eating monkey, in the *Bible Historiee* by Antoine Verard. The original woodcut was recycled in the illustrations of many subsequent publications—including this paragraph—effectively cementing monkeys in the Gothic tradition, and becoming a fixture in the Fall of Man, a Christian doctrine that describes the human transition from a state of innocence to one of disobedience and sin.

But how did monkeys come to symbolize temptation? Curiosity is the essential ingredient of temptation, a connection exemplified in a twelfth century sculptural decoration of Virtues and Vices at Chartres Cathedral. Curiosity was an established vice in Early Christian theology, sometimes represented as the lowest step on the ladder of Pride. The art historian Horst Janson argued that monkeys were innately curious objects of curiosity, a combination of traits that was simultaneously fascinating and repulsive. This dual meaning of curiosity could partly explain how scrumping monkeys became a visual allegory. They embody the transition between innocence and sin.

Exemplary masterworks include *Adam and Eve* by Ludwig Krug in 1514 and *Earthly Paradise with the Fall of Man* by Rubens and Brueghel in ca. 1615. Both works create a strong contrast between scrumping monkeys and the crafty serpent. Monkeys are never malicious; they exist as a visual metaphor for curiosity, temptation, and sin, the same traits that led to Adam's undoing. Janson (1952) put it this way, “[fruit-eating monkeys] are a projection of man's own weakness” (p. 133). The Irish humourist Oscar Wilde is noted for saying, “life imitates art far more than art imitates life.” Epitomizing this view is the word *thagomizer* (the caudal spikes of stegosaurid dinosaurs), which cartoonist Gary Larson coined in 1982. It is now widespread in the scientific literature. The present use of *scrumping*, however, is a case of life imitating art imitating life.

**Figure 1.**
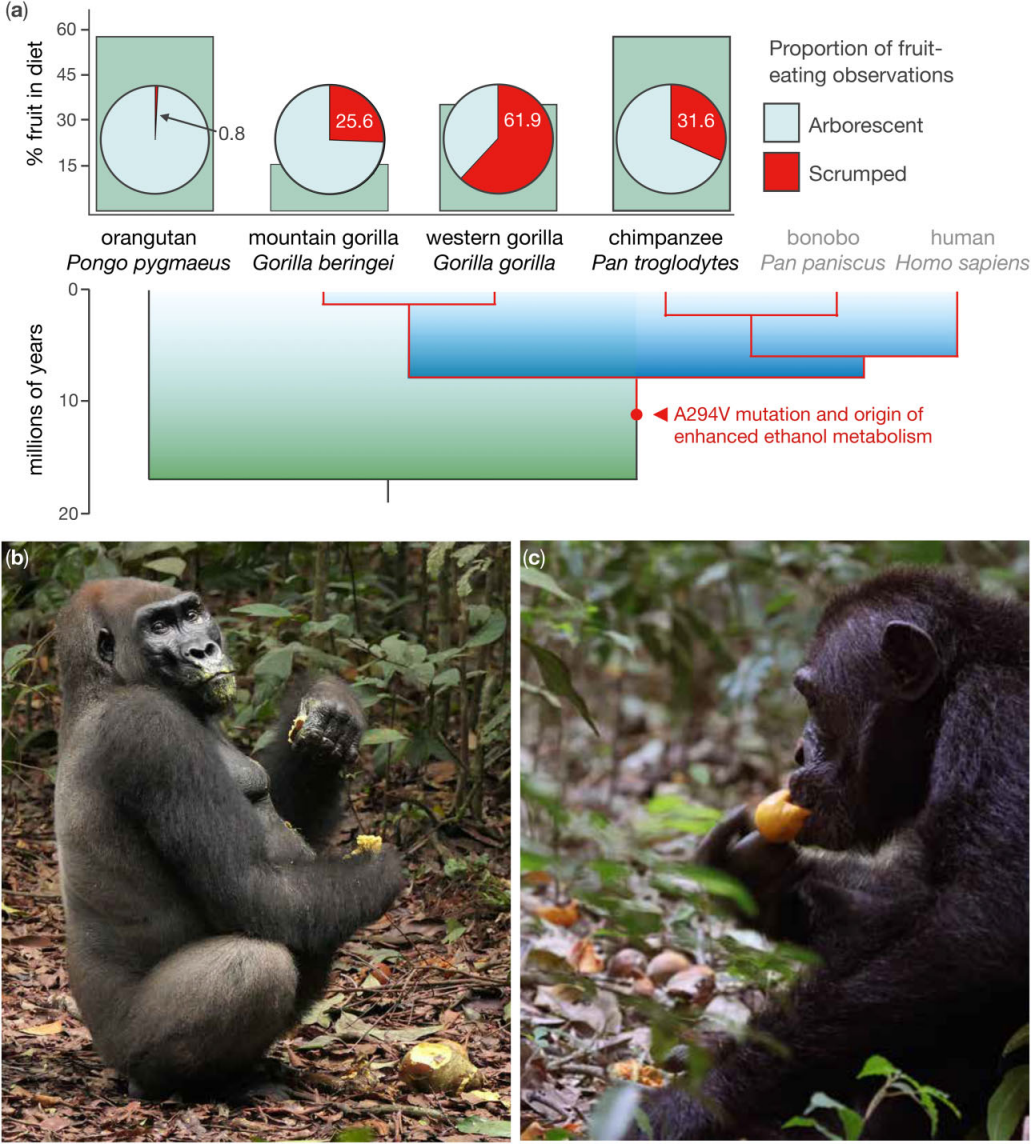
The (a) frequency of frugivory is broadly comparable across studies of orangutans (*Pongo pygmaeus wurmbii*; Vogel et al. 2015; *n* = 5553 hours of fruit-feeding), western gorillas (*Gorilla gorilla gorilla*; Robbins et al. 2022; *n* = 16,696 scans of fruit-feeding), and eastern chimpanzees (*Pan troglodytes schweinfurthii*; Villioth et al. 2023; *n* = 638 hours of fruit-feeding), and it remains important, if less so, for mountain gorillas (*Gorilla beringei beringei*; Ostofsky and Robbins 2020; *n* = 25,590 scans of fruit-feeding), but this traditional approach to quantifying diet obscures striking differences in fruit-feeding at arborescent heights versus ground level, or *scrumping*. Drawing attention to this distinction highlights the importance of scrumping to African apes, species that share the A294V mutation of *ADH7* (also referred to as *ADH4*), the gene that encodes the alcohol dehydrogenase class IV (ADH4) enzyme (Pinto et al. 2023). This single amino acid change accounts for a fortyfold increase in catalytic efficiency. (b) Scrumping of *Pentadesma butyracea* (Clusiaceae) by a western gorilla; photograph: MMR. (c) Scrumping of *Gambeya albida* (syn. *Chrysophyllum albidum*; Sapotaceae) by an eastern chimpanzee; photograph: CH.

Equipped with this word, we were curious to quantify the frequency of scrumping among great apes. We surveyed dietary reports for orangutans (*Pongo pygmaeus*), western gorillas (*Gorilla gorilla*), mountain gorillas (*Gorilla beringei*), and chimpanzees (*Pan troglodytes*), cross-referencing fruit-feeding observations with the vertical height of the focal animal or group. If a given fruit develops in the middle or upper canopy levels, and if feeding occurred at ground level (0 meters), then we classified the behavior as scrumping. This exercise revealed divergent patterns, with broadly comparable levels of frugivory obscuring stark differences in scrumping (figure 1a). African apes are regular scrumpers, bolstering Carrigan and colleagues’ (2015) hypothesis, but we don't know if it exposes them to meaningful levels of ethanol, as was predicted by Bowland et al. (2025a). It is an empirical question that commands attention.

## Last call

**Figure 2.**
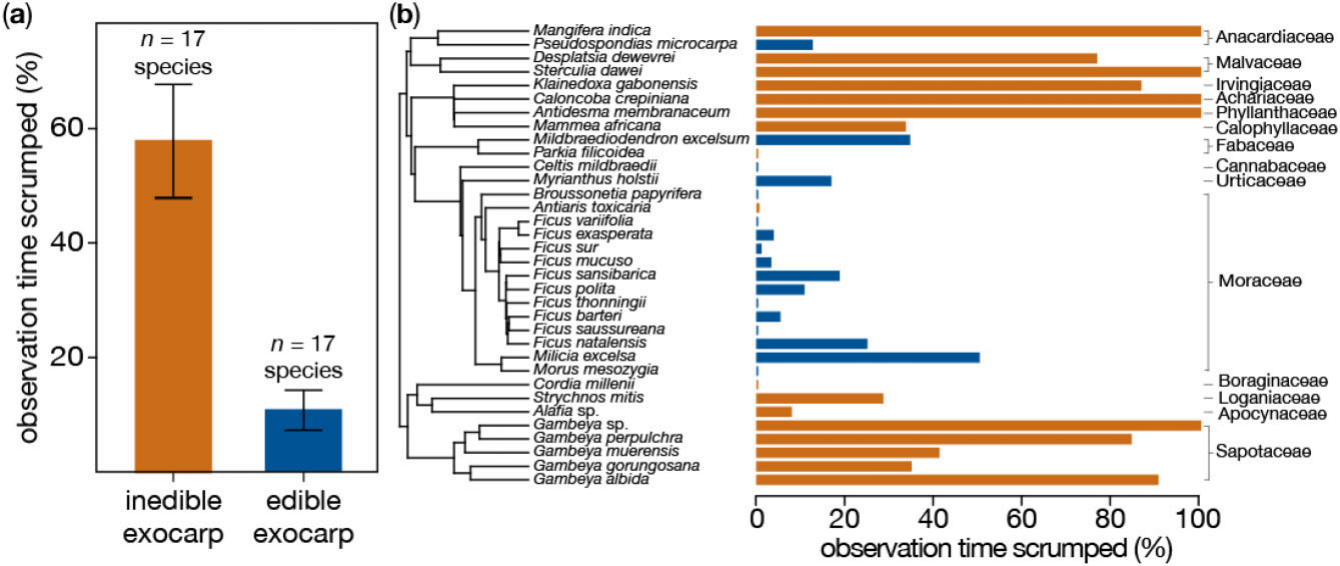
The chimpanzees of Budongo, Uganda, tend to (a) scrump fruits with inedible exocarps (*t*(*19.9*) = 4.51, *p* = .0002; *n* = 638 hours of fruit-feeding observations). The (b) phyletic distribution of scrumping frequency, color coded by exocarp edibility. Covariation of these variables was clustered among closely related taxa: the maximum likelihood estimate of Pagel's *λ* for the residual error of the correlation was 1.08, indicating far greater association than expected under a Brownian motion model of evolution (Revell and Harmon 2022, Pearse et al. 2025).

Buoyed by these findings, we reflected on the types of fruits that elicit scrumping. The foremost trait that sprang to mind was an inedible exocarp—a fruit's outermost protective tissue (skin, peel, husk, etc.; figure 2a)—and it is tempting to ask whether thicker or tougher exocarps evolved to deter moldering pathogens on the damp forest floor. Slowing fungal–bacterial rot is expected to benefit fermenting yeasts and prolong the allure of fallen fruits to terrestrial seed dispersers, including African great apes. Our conjecture stems from the arguments of Leighton and Leighton (1983), who described inedible, indehiscent exocarps as primate-adapted traits, and of those Casorso and colleagues (2023), who reported greater ethanol concentrations in mammal-dispersed fruits. It also motivated us to explore the evolutionary relationships in our data set. Using the *phytools* package in R (Revell and Harmon 2022), we plotted the frequency of scrumping across a phylogeny of plant species (Janssens et al. 2020), revealing an extremely high degree of clustering among the scrumped fruits of Budongo, Uganda (figure 2b).

When we expanded our analysis to the full data set of 154 fruit species consumed by orangutans, gorillas, and chimpanzees, we found far less phyletic covariation (Pagel's *λ* = .56) and no relationship between exocarp inedibility and scrumping frequency (*t*(100.3) = –1.24, *p* = .22). Such outcomes reflect the limits of relying on feeding behavior as a crude proxy for the physical traits of exocarps; orangutans rarely scrump, and gorillas are essentially indifferent to fruit exocarps, eating them whole. A proper analysis of fruit exocarps and their functional and evolutionary ecology may prove (ahem) fruitful.

## Closing time

Scrumping frugivores are probably ingesting highly variable concentrations of ethanol, ranging from negligible to moderate levels, depending on the plant species and stage of ripeness when a given fruit reaches the ground (Bowland et al. 2025b). Measuring this variation is crucial to evaluating the potential selective advantages of scrumping for African apes. For example, there is a caloric reward from the ethanol itself, not to mention the energy savings of *not* climbing a fruiting tree, a hazardous activity (Kraft et al. 2014). Scrumping may also mitigate feeding competition. Arboreal monkeys are unapologetic consumers of unripe fruits, exploiting a critical resource during an early stage of development. Such temporal competition is thought to have exerted a strong selective pressure on the cognitive faculties of apes, including tool-use and careful route-planning (Rosati 2017). Scrumping with a turbocharged ADH4 may have given African apes a similar advantage, yielding access to fruit resources beyond the temporal window preferred by monkeys. Indeed, two of the anatomical hallmarks of apes—their spatulate incisors and greater manual dexterity (Lucas et al. 2008, Dominy et al. 2016)—are essential tools for assessing or removing fruit exocarps during scrumping. So it is tempting to ask whether scrumping was instrumental to the evolution and success of crown African apes, including the earliest hominins.

Finally, scrumping could foster social opportunities. Cofeeding, and even proactive sharing of high-value foods, including fruit, is common among apes (Samuni et al. 2018), whereas human co-consumption of alcohol is often integral to feasting and sacred rituals, events that produce and reinforce community identity and cohesion. Is it possible to trace the roots of these human foodways to social scrumping of fermented fruits in the rainforests of Africa (Bowland et al. 2025b)? Exploring this idea will require integrated observational data of scrumping and social behavior. How does sharing fermented fruits shape the formation and maintenance of social bonds? Does sharing produce social capital for specific individuals, affecting power structures and social relationships? Could regular scrumping influence long-term group stability? Connecting these dots may seem far fetched, but now we have a word to consider the possibility.

## Author Biography

Nathaniel J. Dominy (nathaniel.j.dominy@dartmouth.edu) is affiliated with the Department of Anthropology and the Department of Biological Sciences and Luke D. Fannin is affiliated with the Department of Anthropology and the Graduate Program in Ecology, Evolution, Environment, and Society, both at Dartmouth College, in Hanover, New Hampshire, in the United States. Erin R. Vogel is affiliated with the Department of Anthropology, Center for Human Evolution Studies, at Rutgers, the State University of New Jersey, in New Brunswick, New Jersey, in the United States. Martha M. Robbins is affiliated with the Department of Primate Behavior and Evolution at the Max Planck Institute for Evolutionary Anthropology, in Leipzig, Germany. Catherine Hobaiter (clh42@st-andrews.ac.uk) is a primatologist affiliated with the School of Psychology and Neuroscience at the University of St Andrews, in St Andrews, Scotland, in the United Kingdom.
